# A comprehensive phylogenetic analysis of copper transporting P_1B_ ATPases from bacteria of the *Rhizobiales* order uncovers multiplicity, diversity and novel taxonomic subtypes

**DOI:** 10.1002/mbo3.452

**Published:** 2017-02-20

**Authors:** Ciro Cubillas, Fabiola Miranda‐Sánchez, Antonio González‐Sánchez, José Pedro Elizalde, Pablo Vinuesa, Susana Brom, Alejandro García‐de los Santos

**Affiliations:** ^1^ Programa de Ingeniería Genómica Centro de Ciencias Genómicas Universidad Nacional Autónoma de México Cuernavaca Morelos México

**Keywords:** ATPases, CopA, copper, phylogeny, *Rhizobiales*

## Abstract

The ubiquitous cytoplasmic membrane copper transporting P_1B‐1_ and P_1B‐3_‐type ATPases pump out Cu^+^ and Cu^2+^, respectively, to prevent cytoplasmic accumulation and avoid toxicity. The presence of five copies of Cu‐ATPases in the symbiotic nitrogen‐fixing bacteria *Sinorhizobium meliloti* is remarkable; it is the largest number of Cu^+^‐transporters in a bacterial genome reported to date. Since the prevalence of multiple Cu‐ATPases in members of the *Rhizobiales* order is unknown, we performed an *in silico* analysis to understand the occurrence, diversity and evolution of Cu^+^‐ATPases in members of the *Rhizobiales* order. Multiple copies of Cu‐ATPase coding genes (2–8) were detected in 45 of the 53 analyzed genomes. The diversity inferred from a maximum‐likelihood (ML) phylogenetic analysis classified Cu‐ATPases into four monophyletic groups. Each group contained additional subtypes, based on the presence of conserved motifs. This novel phylogeny redefines the current classification, where they are divided into two subtypes (P_1B‐1_ and P_1B‐3_). Horizontal gene transfer (HGT) as well as the evolutionary dynamic of plasmid‐borne genes may have played an important role in the functional diversification of Cu‐ATPases. Homologous cytoplasmic and periplasmic Cu^+^‐chaperones, CopZ, and CusF, that integrate a CopZ‐CopA‐CusF tripartite efflux system in gamma‐proteobacteria and archeae, were found in 19 of the 53 surveyed genomes of the *Rhizobiales*. This result strongly suggests a high divergence of CopZ and CusF homologs, or the existence of unexplored proteins involved in cellular copper transport.

## 1 Introduction

Copper became bioavailable about one billion years ago when the rise of O_2_ caused a loss of soluble iron and an increase in soluble copper (Cu^2+^) (Rubino & Franz, 2012). Since then, organisms have evolved a diversity of mechanisms to deal with fluctuating copper concentrations in their environments. Copper is a two‐edged sword: it is an essential micronutrient for most organisms that synthesize cuproenzymes, but it is highly toxic when intracellular copper levels surpass trace concentrations (Dupont, Grass, & Rensing, 2011). Excessive copper destabilizes Fe‐S clusters, competes with other metals for protein binding sites and may catalyze the formation of reactive oxygen species (Macomber & Imlay, 2009). The cytoplasmic homeostasis of Cu^+^/Cu^2+^ is maintained by transmembrane proteins belonging to the P_1B_ type ATPase family, widespread in all life domains, which pump Cu^+^/Cu^2+^ out of the cytoplasm employing active transport (Chan et al., 2010; Palumaa, 2013; Sazinsky et al., 2007; Singleton & Le Brun, 2007). This Cu^+^‐ATPase, known in the vast majority of bacteria as CopA, has also been called CopB, CopF, ActP, or CtpA. In this article, we will use CopA to designate Cu‐ATPases, to avoid confusion in the nomenclature. The typical CopA protein contains eight transmembrane helices (TMH), a soluble nucleotide binding domain (N) and a phosphorylation domain (P). The seminal study on the structural and functional diversity of P_1B_ type‐ATPases classified CopA transporters into two subtypes P_1B‐1_ and P_1B‐3_ (Fig. S1) (Argüello, 2003). Subgroup P_1B‐1_ contains archaeal, prokaryotic, and eukaryotic Cu^+^/Ag^+^ transporters. The amino acid motifs involved in metal binding and translocation are two cysteine residues (CXC) in TMH6, a tyrosine and asparagine (YN) residues in TMH7 and methionine and serine residues in TMH8 (MXXSS). P_1B‐1_ ATPases also share N‐terminal MBDs of 60–80 amino acids, which are usually rich in metal‐binding residues. This domain contains a CXXC motif, which can be repeated more than once (Fig.S1) (Argüello, 2003; Argüello, Eren, & Gonzalez‐Guerrero, 2007). The P_1B‐3_ subtype clusters bacterial and archaeal Cu^2+^ transporters that share the CPH (TMH6) motif, crucial for binding Cu^2+^, the GYN(X)_4_ P motif in TMH7 and a MSXST motif in TMH8. They also share an N‐MBD of 30–100 amino acids, containing H‐rich stretches (Fig. S1). This classification was recently confirmed through similarity‐based methods of protein clustering (Smith, Smith, & Rosenzweig, 2014).

Thorough biochemical studies in *Archaeoglobus fulgidus* and *E. coli* have demonstrated that the efflux of cytoplasmic copper to the periplasm requires the interaction of Cu‐chaperones‐Cu‐ATPases, CopZ‐CopA, and CopA‐CusF, (Gonzalez‐Guerrero & Argüello, 2008; Padilla‐Benavides, George Thompson, McEvoy, & Argüello, 2014) (Fig. S2). The CopZ copper chaperone is a cytoplasmic protein that binds Cu^+^ and delivers it to CopA. Then CopA delivers copper to the periplasmic CusF chaperone, which finally transfers copper to the CusCFBA efflux system (Franke, Grass, Rensing, & Nies, 2003) that pumps copper out of the cell. The absence of a typical CopZ chaperone in *Streptococcus pneumoniae* led to the identification of an alternative Cu‐chaperone, named CupA, capable of transferring Cu^+^ to CopA (Fu et al., 2013).

The *Rhizobiales* order groups a variety of families of alpha‐proteobacteria with agronomic and medical relevance. Members of some families are facultative symbiotic diazotrophs that can be found either as free‐living organisms in the rhizosphere or as intracellular symbionts in root nodules of leguminous plants, besides being plant or animal pathogens (Carvalho, Souza, Barcellos, Hungria, & Vasconcelos, 2010) (Table 1). Rhizobia, as other soil bacteria, may be exposed to high concentrations of copper ions as a consequence of the Cu‐based fungicides and bactericides used in the crop fields, and during the plant or animal‐pathogen interaction, as result of the Cu provided by the host in response to infections (Chaturvedi & Henderson, 2014; Cheruiyot, Boyd, & Moar, 2013; Fu, Chang, & Giedroc, 2014).

**Table 1:** Members of the *Rizobiales* order analyzed in this study

Genera	Number of species	Number of strains	Habitat or metabolism	Range of chromosomes number	Range of plasmids number
*Agrobacterium*	4	4	Plant Pathogen	1–2	2
*Aureimonas* (formely *Aurantimonas*)	1	1	Potential human pathogen and different environmental sources	1	8
*Bartonella*	2	2	Mammalian pathogens	1	1–2
*Beijerinckia*	1	1	Free‐living in soil	1	2
*Bosea*	1	1	Thiosulfate‐oxidizing/plant associated	1	1
*Bradyrhizobium*	1	1	Facultative symbiotic diazotrophs	1	1
*Brucella*	5	5	Mammalian pathogens	2	0
*Chelativorans*	2	2	EDTA‐degrading	1	3
*Ensifer*	1	1	Facultative symbiotic diazotrophs	2	2
*Hoeflea*	1	1	Marine Iron‐oxidizing	1	2
*Martelella*	1	1	Halophyte	1	2
*Mesorhizobium*	2	2	Facultative symbiotic diazotrophs	1	1–2
*Methylobacterium*	5	7	Methylotrophic plant‐associated	1	1–8
*Neorhizobium*	1	1	Facultative symbiotic diazotrophs	1	2
*Nitrobacter*	1	1	Nitrite‐oxidizing free‐living in soil	1	3
*Ochrobactrum*	1	1	Opportunistic human pathogen	2	4
*Oligotropha*	1	2	Wastewater CO‐utilizing	1	1–2
*Pelagibacterium*	1	1	Seawater halotolerant	1	1
*Rhizobium*	3	7	Facultative symbiotic diazotrophs	1	1–6
*Rhodopseudomonas*	1	1	Photosynthetic, nitrogen and carbón fixer	1	1
*Shinella*	1	1	Degradation of toxic aromatic compounds	1	12
*Sinorhizobium*	3	8	Facultative symbiotic diazotrophs	1	1–4
*Xanthobacter*	1	1	Chemolitho‐autotrophic free‐living diazotroph	1	1
23	40	53			

The multipartite genomes of most species grouped in the *Rhizobiales* order are characterized by the presence of one or two chromosomes, megaplasmids and numerous large plasmids, some of them transmissible by conjugation (Jumas‐Bilak, Michaux‐Charachon, Bourg, Ramuz, & Allardet‐Servent, 1998; MacLean, Finan, & Sadowsky, 2007) (Table 1). This may have played an important role in the spread, acquisition, and evolution of metal resistance genes. Bacterial plasmids have been shown to be critical in bacterial Cu tolerance, and are highly mobile among strains growing in metal polluted soils (Cooksey, 1990; Lakzian, Murphy, & Giller, 2007; Monchy et al., 2007; Tetaz & Luke, 1983). Functional plasmid‐encoded CopA transporters have been reported for three rhizobial species (Landeta et al., 2011; Reeve, Tiwari, Kale, Dilworth, & Glenn, 2002). The presence of multiple Cu‐ATPase coding genes in *S. meliloti,* highlighted in the pioneer study on functional classification of P‐type ATPases, led to the physiological characterization of five plasmid‐encoded Cu‐ATPases present in this organism (Argüello, 2003; Argüello et al., 2007; Patel, Padilla‐Benavides, Collins, & Argüello, 2014; Smith et al., 2014). This study revealed a nonredundant physiological role that is important for saprophytic and symbiotic lifestyles, as well as the presence of a novel CopA regulated by redox stress. A cladogram inferred from a clustalW alignment grouped these five CopA homologs into three different clades but nothing is known about their evolutionary relationships to other rhizobial and nonrhizobial Cu‐ATPases (Patel et al., 2014). Also, a genome‐wide study on trace element utilization revealed that two *Sinorhizobuim* species contain uncommonly large cuproproteomes, which may be related to the numerous CopA transporters and other trafficking proteins (Zhang & Gladyshev, 2010).

Several questions arise from the studies in *S. meliloti:* Is the model of five plasmid‐encoded Cu‐ATPases the archetype of Cu‐ATPases for the *Rhizobiales* order? How diverse are the multiple Cu‐ATPase homologs encoded in the *Rhizobiales* genome? To what extent has horizontal gene transfer (HGT) contributed to Cu‐ATPases acquisition? Additionally, we are also interested in analyzing the occurrence of the CopZ/CupA and CusF homologs, cytoplasmic, and periplasmic copper chaperones, respectively, which might be associated with the multiple Cu^+^‐ATPases.

To answer these questions, we surveyed the occurrence of CopA, CopZ/CupA and CusF homologs encoded in members of the *Rhizobiales* order with multi‐replicon genomes and analyzed the diversity of CopA homologs through a maximum‐likelihood (ML) phylogenetic analysis.

Our study revealed that *copA* multiplicity is a highly conserved characteristic in the genomes of *Rhizobiales*. The diversity of these CopA transporters is higher than previously assessed and is influenced by horizontal gene transfer. We propose a novel phylogeny that distributes bacterial Cu‐ATPases in four monophyletic group and six subtypes based in conserved motifs (P_1B‐1_, P_1B‐1a_, P_1B‐1b_, P_1B‐1c_, P_1B‐3_, P_1B‐3a_). The complete CopZ‐CopA‐CusF set of proteins potentially involved in cytoplasmic copper efflux was only found in 19 of the 53 analyzed genomes of *Rhizobiales*.

Although this bioinformatic analysis was focused on P_1B_ ATPases, our results on multiplicity and diversity of metal transporters have general implications regarding the evolution of resistance, adaptation, and innovation in bacteria.

## 2 Methods

### 2.1 Data set, multiple sequence alignments and phylogenetic analysis

As mentioned above, the five Cu‐ATPases of *S. meliloti* 2011 are encoded in two extrachromosomal replicons (Patel et al., 2014). Plasmids usually have a higher copy number than chromosomes, which may result in higher expression levels of plasmid‐encoded genes, representing an advantage for this bacterium. The increased copy number of plasmids might also accelerate gene diversification (San Millan, Escudero, Gifford, Mazel, & MacLean, 2016; Sano, Maisnier‐Patin, Aboubechara, Quiñones‐Soto, & Roth, 2014). To widen our knowledge regarding these metal transporters, we surveyed the occurrence of CopA, CopZ/CupA and CusF homologs in 53 fully sequenced multireplicon genomes of rhizobia species with different lifestyle or metabolism (Table 1).

To test if the model of five plasmid‐encoded Cu‐ATPases reported for *S. meliloti* 2011 is the archetype for the *Rhizobiales* order, we collected 313 P1B‐ATPases sequences from the Pfam family PF00122 (Table S1) belonging to 53 fully sequenced multireplicon genomes of species grouped in the *Rhizbiales* order with different lifestyle or metabolism (Table 1). The construction of the data set and the different steps of the phylogenetic analysis are depicted in Fig.S3. The P_1B_‐ATPases and Cu‐Chaperones (CopZ/CupA and CusF) homologs were retrieved from their respective Pfam families (CopA, PF00122; CopZ, PF00403; CupA, PF13473; and CusF, PF11604) either filtered by using species–specific tags (e.g., *Rhizobium* copper‐translocating/chaperones) or using the profile Hidden Markov Model (HMM) for each family as query in hmmsearch (cut‐off value 10^−3^, under default settings) for those genomes whose proteins have not been assigned yet to specific protein families (i.e., *Rhizobium tropici*). Other sequences were retrieved from PATRIC (Bacterial Bioinformatic Resource Center, Wattam et al., 2014). The P_1B_‐type‐ATPases, CopZ/CupA, and CusF homologs found in the 53 genomes belonging to the *Rhizobiales* order are shown in supplementary tables [Link], [Link], [Link], [Link]. These P_1B_‐type‐ATPases may have as substrate one or more of the following metals: Cu^+^, Ag^+^, Cu^2+^, Zn^2+^, Co^2+^, Pb^2+^, Cd^2+^, and Mn^2+^.

To infer the phylogeny, the data set of 313 P_1B_ ATPases was complemented with 45 nonrhizobia, characterized P_1B_ ATPases whose metal(s) specificities have been assessed experimentally (Table S1). Thirty five K^+^ transporters grouped in ATPase family P_1A_ were included as out‐group. Full‐length multiple sequence alignments were done using MUSCLE and hmmalign. Since the profile HMM for the P_1B_ ATPases family available at Pfam lacks most critical motifs required for metal transport, including some involved in metal‐ion selectivity (Gonzalez‐Guerrero & Argüello, 2008), we used an *ad hoc* profile HMM constructed with 53 characterized P_1B_ ATPases, using hmmbuild under default parameters. This novel alignment and the resultant new profile HMM contained all relevant regions involved in metal transport (Fig.S4).

To infer the highest likelihood phylogenetic tree for P_1B_ ATPases proteins, we started with 100 random seed trees in addition to a BioNJ tree to perform 101 searches. Tree searching under the ML criterion was performed with PhyML v3.026, using the LG+G+f model as the substitution matrix with gamma‐correction among‐site rate variation. The best tree, shown in Fig. S5, had the highest log‐likelihood score from these 101 searches.

### 2.2 Detection of phylogenetic incongruence and putative horizontal transfer events

The HGT analysis presented in this study was based on the identification of putative recently transferred genes representing bona fide examples of HGT events. Since these genes still maintainmost of the nucleotide composition of their parental hosts, they were searched for through similarity‐based methods, using BLASTP analyses with default parameters.

The 155 Cu‐ATPases of *Rhizobiales* defined by the phylogenetic analysis were used as query to find their closest homologs in the nonredundant NCBI database. Thirty rhizobial Cu‐ATPases transporters had as closest homologs, Cu‐ATPases from species distantly related to members of the *Rhizobiales* order belonging to alpha‐, beta‐ and gamma‐proteobacteria and Bacteroidetes (Table S6). Their foreign origin was also assessed by nucleotide compositional methods, searching for deviations between the sequence composition of such genes and their respective genomes. The G+C content was calculated at http://www.genomicsplace.com/gc_calc.html. Genomic G+C content was obtained from: http://www.ncbi.nlm.nih.gov/genome/browse/.The codon adaptation index (CAI) of *copA* and the expected e‐CAI were calculated at the E‐CAI server (Puigbo, Bravo, & Garcia‐Vallve, 2008). E‐CAI web server provides a threshold value, named eCAI, to discern if the differences in CAI are statistically significant. The eCAI value is calculated by generating 500 random sequences with the same amino acid composition as the query but with randomly assigned codon usage (Puigbo et al., 2008). The normalized CAI is defined as the quotient between the CAI of a gene and its expected e‐CAI value. Values below one indicate a bias in the codon usage preferences of a gene, relative to the codon usage preferences of their respective genomes, used as reference.

The location of *copA* genes in putative genomic islands was predicted by the sequence composition‐based method available at IslandView (pathogenomics.sfu.ca/islandviewer/). Their location into a cluster of metal resistance genes was searched for with Microbial Genomic Contex Viewer (Overmars, Kerkhoven, Siezen, & Francke, 2013; http://mgcv.cmbi.ru.nl/).

The hypothesis that these genes arrived through HGT was tested with a phylogenetic approach, comparing the species’ phylogeny, inferred from 16S rRNA genes, with the phylogeny inferred from homologous Cu^+^‐ATPase coding genes (Fig. S6). Aligments for 16S rRNA genes were built using the secondary‐structure aware Infernal aligner (Nawrocki, Kolbe, & Eddy, 2009) incorporated in the Ribosomal Database website (https://rdp.cme.msu.edu/). The Cu‐ATPases sequences were aligned using an HMMER profile built from 53 characterized Cu‐ATPases proteins. These phylogenies were performed with an ML‐phylogenetic analysis under GTR+I+G model for 16S rRNA and LG+G+f model for putative CopA proteins, using nucleotides and amino acid alignments, respectively. The phylogenies were inferred using a parallel Pthreads‐based version of RAxML v8.2.4, this software supports SSE3 vector instructions (Stamatakis, 2014). The ML‐search started with 1000 random seed trees, the best tree was selected (Figure S6). Rapid bootstrapping (Stamatakis, 2014) was uses to assess the branch support in the tree. The number of necessary replicates was estimated using the extended majority rule criterion, 150 and 1,000 replicates for Cu‐ATPases and 16S rRNA phylogenies, respectively (Pattengale, Alipour, Bininda‐Emonds, Moret, & Stamatakis, 2010).

## 3 Results

### 3.1 A maximum likelihood phylogeny groups Cu‐ATPases into four monophyletic groups

To gain a broader view of the diversity of P_1B_ ATPases present in the 53 multi‐replicon members of the *Rhizobiales* order contextualized within an evolutionary framework, we analyzed 313 P_1B_ ATPases encoded in the multireplicon genomes of 53 strains. Fifty three characterized P_1B_ type ATPases known to transport copper or other metals present in eukaryots, archaea, and other bacteria were also included in the phylogenetic analysis (Table S1). Thirty five P1A‐type KdpB subunits (potassium‐extruding ATPases) and nine putative zinc transport ATPases (ZntA) from different rhizobia were included as out‐groups. To improve the accuracy of previous classifications, a profile‐HMM was built from the alignment of the previously mentioned characterized P_1B_ ATPases, which contain all the structurally conserved regions (Gourdon et al., 2011) (Table S1, Fig S4). We decided to build this new model instead of using the one available at Pfam because the last one does not include the N‐terminal MBD (see Experimental Methods) (Fig S4) and only shows the most conserved region of the P_1B_ TPase family, the actuator and the ATP‐binding domains. The alignment of the total data set (410 P_1B_‐ATPases) with our profile‐HMM was critical to analyze an increased number of amino acid positions, allowing us to obtain a better description of the P_1B_‐type‐ATPase family composition.

In previous studies the P_1B_‐ATPase relationships were inferred from ClustalW2 cladograms (Argüello, 2003; Argüello et al., 2007; Gonzalez‐Guerrero, Raimunda, Cheng, & Argüello, 2010; Patel et al., 2014). The use of *ad hoc* HMM as well as the maximum likelihood method for phylogenetic analysis allowed us to perform a more rigorous and accurate inference of the evolutionary relationships among the 410 P‐type‐ATPases included in our data set (Fig.S5).

Our ML‐phylogenetic analysis classified 181 of the 410 P_1B_ type ATPases as Cu‐ATPases and they were assorted in 4 monophyletic groups (VI, VII, VIII and XIV,) with branch bipartitions well supported by Shimodaira‐Hasegawa‐like ρ value ≥0.9, Each one of the Cu‐defined clades contains at least one characterized Cu‐ATPase. Bacterial Cu‐ATPases not belonging to the *Rhizobiales* order, were assorted in three different clades (I, III and V). Cu‐ATPases from eukaryotes were included in clade IV and the Zn/Cd/Mn/Mg/K‐ATPases, retrieved during the data mining, were grouped in five well supported (ρ value ≥0.9) monophyletic clades (Fig. S5).

### 3.2 The Cu^+^‐ATPase subtypes differ in signature motifs at the N‐terminal MBD and transmembrane domains

Fifty of the 181 putative Cu^+^‐ATPases were grouped in subtype VIII (Fig. S5, Table 2 and Table S5). These copper transporters maintain the invariant signature motifs for P_1B‐1_‐Cu^+^‐ATPases in the transmembrane helices TM6 (CPC) and TM8 (MXXSS) (Fig. S1) previously described (Argüello, 2003). All these Cu‐ATPases conserve two heavy metal associated domains (HMA) and the CASC motif at the cytoplasmic N‐MBD, suggesting that they may have functional relevance (Table 2).

**Table 2:** Polymorphisms within conserved motifs N‐MBD, TM 6, 7, and 8 of rhizobial Cu‐ATPases subfamilies

Conserved motifs					
Group (Number of Cu‐ATPases)	N‐MBD (CXXC)	TM6	TM7	TM8	Subtype
VIII (50)	CASC CASC CASC	CPC CPC CPC	YN(X)_4_P YN(X)_4_P YN(X)_4_P	MALSS MAMSS MAFSS	IB‐1^a^
VIa (6) VIb (5) VIc (3)	H‐Rich H‐Rich Poor H content	CPH CPH CPD	YN(X)_4_P YN(X)_4_P YN(X)_4_P	MSAST MSAST MSGSS	IB‐3^a^ IB‐3^a^ IB3‐a^b^
VII (25) VII (14) VII (15)	H‐Rich/ CPIC CPIC H‐Rich/CPKC	CPC CPC CPC	YN(X)_4_P YN(X)_4_P YN(X)_4_P	MSLSS MALSS MSLSS	IB‐1a^b^ IB‐1^a^ IB‐1b^b^
XIV (56)	CAGC CAAC CASC CAAC	CPC CPC CPC CPC	YN(X)_4_P YN(X)_4_P YN(X)_4_P YN(X)_4_P	MSGSS MSGSS MSGSS MSLSS	IB‐1c^b^ IB‐1c^b^ IB‐1c^b^

Parenthesis indicates number of Cu‐ATPases in each group.

a Established subtype classification (Argüello et al., 2007).

b Novel subtypes (This study).

The few typical P_1B‐3_‐Cu^2+^‐ATPases contained in our data set, identified as Cu^2+^ transporters (Fig. S5), containing an N‐terminal rich in His residues and the invariant CPH motif in TM6, were located in group VI (Table 2 and Table S5 Cu‐ATPase subtypes). Members of this group could be distributed in three subtypes based in the presence of different motifs and well supported bipartitions (Table S5 Cu‐ATPases subtypes). Cluster VIa groups the poorly characterized Cu^2+^‐ATPases, HRA‐1 and HRA‐2, from *E. coli, Staphylococcus aureus* CopB, and *Thermus thermophilus* 1371. The location of *Enterococcus hirae* CopB in this cluster is intriguing because of its ability to transport Cu^+^ (Solioz & Odermat, 1995). However, its ion transduction region presents the CPH signature motif invariable in P_1B‐3_‐Cu^2+^‐ATPase (Odermatt, Suter, Krapf, & Solioz, 1993). The imidazol from the His residue forms a stronger bond with Cu^2+^ than Cu^+^ (Argüello, Gonzalez‐Guerrero, & Raymunda, 2011).

Subgroup VIb groups six putative Cu^2+^‐ATPases from the EDTA–degrading *Chelativorans* sp, the osmotolerant *Pelagibacterium halotolerans,* the crude‐oil‐degrading bacterium *Chelatococcus sp* CO‐6 and the seawater halotolerant *Hoeflea sp*. IMCC 20628 together with the characterized archaeal CopB from the hyperthermophilic *Archaeoglobus fulgidus* known to transport Cu^2+^ (Mana‐Capelli, Mandal, & Argüello, 2003). Interestingly, their closest homologs, by means of BLAST comparisons, retrieved ATPases from extremophile bacteria with thiosulfate oxidation capabilities as well as methanogenic and osmotolerant *Archaea*. Both clusters contain Cu^+^‐ATPases with poly His at the N‐terminal as well as the invariant CPH at TMH6 (Table 2).

The third subtype, VIc, includes three atypical rhizobial Cu‐ATPases from *Xanthobacter autotrophicus*,* Nitrobacter hamburgensis*, and *Bradyrhizobium sp,* which present few histidines at the N‐terminal, contain a novel CPD motif instead of the invariant CPH motif in TM6 and are at least 80 kb longer than Cu‐ATPases from clusters VIa and VIb. This subtype was named P_1B_‐3a (Table 2 and Table S5 Cu‐ATPase subtypes).

Sixty one of the 181 Cu‐ATPases were classified in clade VII; interestingly, these transporters share motifs from both P_1B‐3_ and P_1B‐1_‐Cu^+^‐ATPases (Table 2 and Table S5 Cu‐ATPase subtypes). These hybrid characteristics have not been previously reported. Similar to the characterized Cu‐ATPases from *Legionella pneumophila* (Lpg1024) and *Salmonella typhimurium* (SilP), belonging to this subtype, 25 rhizobial homologs contain a His‐rich extension at the N‐terminal MBD characteristic of subgroup P_1B‐3._ Like the P_1B‐1_ subgroup, they present CPIC as the invariant CXXC motif at N‐terminal MBD as well as a CPC motif at TM 6, instead of the invariant CPH motif distinctive of P_1B‐3_‐Cu^+^‐ATPases (Table 2 and Table S5 Cu‐ATPase subtypes). Following the already established subgroup nomenclature (Argüello, 2003) these transporters were named as P_1B‐1a_‐Cu^+^‐ATPases.

Fifteen of the 61 putative rhizobial Cu‐ATPases clustered in group VII have the largest N‐MBD found in *Rhizobiales*: 160 amino acids preceding the highly conserved CPKC motif. The first 20 amino acids contain a short H‐rich segment of six to 10 residues and two copies of the TRASH/YHS‐domain located downstream of this poly‐H labeled as Ribo previously reported by Ettema, Huynen, de Vos, and van der Oost (2003), which contains a CXCXC signature that might be involved in copper sensing, trafficking and resistance (Table S5). The Cu‐ATPases from *Chelativorans sp* and *M. loti* MAFF303099 lack the CPKC motif; instead, they have a CPIC similar to that from P_1B‐1a_‐Cu^+^‐ATPases. The motifs at TM 6 (CPC), 7 (YN(X)_4_P) and 8 (MSLSS) are highly conserved among P_1B‐1_‐Cu^+^‐ATPases of members of the *Rhizobiales* order (Table 2). Since members of this subtype maintain the CPC motif typical of P_1B‐1_‐Cu^+^‐ATPases, these proteins will be named P_1B‐1b_‐Cu^+^‐ATPases (Table 2).

Clade XIV groups FixI proteins (Fig. S1, Table 2 and Table S5 Cu‐ATPase subtypes). Analysis of *S. meliloti fixI1* and *fixI2* mutants revealed that these Cu‐ATPases do not contribute to the copper tolerance of rhizobia in the free‐living state (Patel et al., 2014). Since FixI proteins are encoded close to high oxygen‐affinity‐cbb3‐type cytochrome oxidases expressed under microaerobic conditions during symbiotic nitrogen fixation (Preisig, Zufferey, Thöny‐Meyer, Appleby, & Hennecke, 1996), it has been hypothesized that FixI1 plays a key role in bacteroid respiration in the microaerobic environment inside the nodule. On the other hand, FixI2 is a housekeeping Cu^+^‐ATPase involved in metallation of a constitutive cytochrome oxidase required for respiration in both, symbiosis and in the free‐living state (Patel et al., 2014). A cladogram inferred from a ClustalW2 alignment indicated that the FixI transporters belong to different clusters (Patel et al., 2014). In our ML‐phylogeny we found small subgroups of FixI, FixI1 and FixI2 spread throughout the clade. Most of the FixI proteins share common N‐MB, TM6, TM7 and TM8 motifs. Since these motifs differs from those present in Cu‐ATPases of clades VI, VII and VIII these Cu‐ATPases subtypes were named P_1B‐1c._ The eukaryotic and prokaryotic ATPases predicted to transport metals other than Cu, were distributed in five different monophyletic clades: IX, (Cd, Co, Zn), X (Ca and Mg), XI (K), XII (Cd and Zn) and XIII (Mn, Zn, and Cd). These transporters will not be described in this study.

### 3.3 Occurrence of Cu‐ATPase subtypes in the multipartite genomes of rhizobia surveyed

The distribution and abundance of the established and novel CopA subtypes in the 53 genomes analyzed in this study are shown in detail in Table S8 and a summary of the data is presented in Tables 3 and 4. The number of Cu‐ATPases per genome is very variable (Table 3), it ranges from zero in two species of the intracellular *Bartonella* pathogens to 8 in the genome of *Rhizobium leguminosarum* bv. viciae 3841. Five Cu‐ATPases subtypes, the number of ATPases reported in the genome of *S. meliloti* 2011 and characterized by Patel et al. (2014), was only found in 5 other rhizobia. Forty three percent of Cu‐ATPases homologs are encoded in plasmids and fifty seven percent in chromosomes (Table S5).

**Table 3:** Number of Cu‐ATPases coded in the 53 multipartite rhizobial genomes surveyed

Number of Cu‐ATPases	Occurrence in rhizobia genomes
0	2
1	11
2	8
3	11
4	8
5	5
6	4
8	2

**Table 4:** Occurrence of Cu‐ATPases subtypes in the 53 rhizobial genomes surveyed

Cu‐ATPases subtypes	Genomes harboring Cu‐ATPases subtypes (%)
P1B‐1	88
P1B‐1a	77
P1B‐1b	36
P1B‐1c	94
P1B‐3	13
P1B‐3a	6

We also analyzed the prevalence and multiplicity of the previously established ATPases subtypes P_1B‐1_ and P_1B‐3_ (Argüello et al., 2007), in the 53 genomes surveyed (Table 4). The P_1B‐1_ subtype was the most frequent Cu‐ATPase, it was found in 88% of the genomes analyzed. Such genomes contain at least one P_1B‐1_‐ATPas. Most of these ATPases (74%) were encoded in the chromosome. In contrast, the P_1B‐3_ subtype was only found in 13% of the 53 surveyed genomes. Among the novel subtypes found in this study, the P_1B‐1a_ was the most frequent, 77% of the genomes contain at least one copy (Table 4 and Table S8).

### 3.4 The diversity of Cu‐ATPase subtypes of the *Rhizobiales* order is influenced by horizontal gene transfer events among alpha‐, beta‐, and gamma‐proteobacteria

Attempting to explain the multiplicity and high diversity of Cu‐ATPase subfamilies in rhizobia, we examined the role that HGT may have played in the acquisition and divergence of Cu‐ATPases in the *Rhizobiales* order. We focused on the identification of recently transferred genes representing bona fide HGT events. Since these genes still conserve most of the nucleotide composition of their parental hosts, they could be detected through BLASTP‐based searches using the 155 Cu‐ATPase sequences as query to find their closest homologs in the nonredundant NCBI database (Zhaxybayeva, Gogarten, Charlebois, Doolittle, & Papke, 2006). Genuine events of HGT are difficult to assess when searching for anciently transferred genes, due to amelioration, the process by which the nucleotide composition (G+C, codon bias, nucleotide frequency) of the putative foreign genes change by the mutational pressure of the new host and over time the foreign genes are indistinguishable of the DNA composition of the host genome. Since the signs of amelioration are not easily identified, the search for ancient acquired genes increases the probability of identifying false laterally transferred genes.

30 Cu‐ATPases, belonging to different *Agrobacterium, Methylobacterium, Ochrobactrum, Oligotropha, Pelagibacterium*,* Chelativorans, Aureimonas, Bosea, Hoeflea*, and *Martelella* species, retrieved as the closest homologs (identity >50%, similarity >70% and coverage >90%) putative Cu‐ATPases encoded in the genome of species distantly related to members of the *Rhizobiales* order (Table S6 HGT). These homologs were found in the genomes of gamma‐proteobacteria (12), beta‐proteobacteria (four), alpha‐proteobacteria other than *Rhizobiales* (13) and Bacteroidetes (one). The high similarity values shared between these rhizobial proteins and their counterparts in beta‐ and gamma‐proteobacteria prompted us to further investigate if they concurred with HGT events.

We found that the G+C content of the 30 Cu‐ATPase coding genes averaged 4.8% points higher than that of their corresponding genome context (Table S7 G+C, CAI and GI). These differences were supported by a *t‐*test (with *p *<* *.00001; at 99% confidence interval following previously reported methods (Hayek, 2013).

Recent laterally transferred genes are expected to have an atypical codon usage in comparison to the codon usage of their genome context. The CAI is the most prevalent method used for analyzing such differences. We used the E‐CAI web server (Puigbo et al., 2008) to quantify the similarity of the codon usage of 30 Cu‐ATPase coding genes compared to the codon usage of their respective genomes. Our analysis revealed that twenty two genes had a normalized value of CAI <1, [CAI/eCAI (*p* < .01)] indicating significant differences in codon usage between the Cu‐ATPases and their genomes, further supporting that those genes may have arrived through HGT (Table S7 G+C, CAI and GI). Four of the genes were predicted to be part of putative detoxification islands by different web tools (Dhillon, Chiu, Laird, Langille, & Brinkman, 2013). Ten putative foreign Cu‐ATPases were localized next to genes coding for diverse metal transporters, transposases and insertion sequences, in clusters whose length ranges from 7 to 75 Kb (Table S7 G+C, CAI and GI).

An ML‐phylogenetic analysis was performed to detect incongruity between the species tree, inferred from the alignment of 16S rRNA genes (Fig. S6A), and the CopA tree inferred from the alignment of the Cu‐ATPAse homologous sequences from the same bacterial species (Fig. S6B). While the 16S rRNA tree classified alpha‐, beta‐, and gamma‐proteobacteria and bacteroidetes in four clearly defined clusters, in the CopA tree several beta‐, gamma‐proteobacteria and bacteroidetes were scattered among the *Rhizobiales* (Fig S2). This phylogenetic incongruence strongly points to HGT as a process that has facilitated the exchange of Cu‐ATPases among distantly related alpha‐, beta‐, and gamma‐ proteobacteria and bacteroidetes.

To get insights into the possible direction of the putative HGT events, the foreing Cu‐ATPases found in rhizobia, homologs of gamma‐proteobacteria, were used as query in BlastP searches, to compare the occurrence of homologs between orders/families of alpha‐ and gamma‐proteobacteria. Whereas BlastP searches of proteins from alpha‐proteobacteria retrieved homologs exclusively from the order *Rhizobiales,* most of them belonging to the *Rhizobiaceae* family, BlastP searches of proteins from gamma‐ proteobacteria retrieved homologs (60% identity, 80% query cover and e‐value = 0) from at least five different orders (*Enterobacterales, Alteromonadales, Xanthomonadales, Vibronales, Pseudomonadales*). The broad distribution of homologs in five gamma‐ proteobacteria orders and the limited occurrence in alpha‐proteobacteria (one order) strongly suggests that these genes come from gamma‐proteobacteria and have been recently transferred to alpha‐proteobacteria.

A similar analysis was carried out with the foreing Cu‐ATPases homologs of beta‐proteobacteria. However, BlastP searches retrieved homologs from four different beta‐proteobacteria orders (*Burkholderiales, Nitrosomonadales, Methylophilales* and *Rhodocyclales*), BlastP searches into alpha‐proteobacteria retrieved homologs exclusively from the *Rhizobiales* order, suggesting that these genes were transferred from beta‐proteobacteria to *Rhizobiales*.

A correlation could not be established among the foreing Cu‐ATPases of rhizobia and their alpha‐proteobacteria homologs other than *Rhizobiales* order. These ATPases were broadly distributed in a variety of genera belonging to *Rhizobiales, Rhodobacterales, Caulobacterales*, and *Sphingomonadales* orders suggesting exchange among them.

The broad occurrence of the putative Cu‐ATPase of *Hoeflea sp* in four of nine orders of alpha‐proteobacteria (*Rhodospirilales, Rhodobacterales, Sphingomonadales*, and *Rhizobiales*) but in two of six orders of *Bacteroidetes* (*Bacteroidetes* order II and *Flavobacterales*) might indicate that this gene has moved from alpha‐proteobacteria to *Bacteroidetes*.

### 3.5 Low occurrence of cytoplasmic and periplasmic copper chaperones homologous to CopZ/CupA and CusF in genomes of the *Rhizobiales*

Most of the CopZ homologs present in the 53 genomes were downloaded from the HMA (PF00403 family, where the functionally characterized CopZ proteins are located. This family, defined by a profile‐HMM, belongs to the Pfam database. The profile HMM and hmmsearch were used to search for CopZ homologs in those genomes whose proteins have not been assigned to a Pfam Database (see Methods for details). These searches are more sensitive than pairwise methods such as BLAST or FASTA, to find distantly related sequences (Eddy, 1998). We found 55 CopZ homologs, 24 coded in plasmids and 31 in chromosomes, distributed in 26 genomes belonging to 10 different genera (Table 5; Table S2 CopZ homologs). The largest number of putative CopZ chaperons was found in the genomes of *Methylobacterium extorquens* AM1 and *Ochrobactrum anthropi* DSM6882 with eight and five homologs, respectively. Similar to previously characterized CopZ proteins, the rhizobial homologs are small proteins varying between 67 and 114 amino acids that adopt the ferredoxin‐like fold. All of them contain the conserved HMA (PF00403) and the signature MTCXXC motif with the two invariable cysteine residues required for binding and transfer of metal ions (Table S2 CopZ homologs).

**Table 5:** Occurrence of putative CopZ,CupA, CopA and CusF proteins in 53 Rhizobial multireplicon genomes^a^

Strains	CopZ	CupA	CusF
*1. Agrobacterium fabrum* str. C58	1C	0	0
*2. Agrobacterium radiobacter* K84	0	0	1C2
*3. Agrobacterium* sp. H13‐3	1C	0	0
*4. Agrobacterium vitis* S4	1P	0	0
*5. Aureimonas sp. AU20*	1	0	0
*6. Bartonella grahamii* as4aup	0	0	0
*7. Bartonella tribocorum* CIP 105476	0	0	0
*8. Bosea sp. PAMC 26642*	0	0	0
*9. Beijerinckia indica* subsp. indica ATCC 9039	0	2C	0
*10. Bradyrhizobium* sp. BTAi1	0	2C	0
*11. Brucella abortus* bv. 1 str. 9‐941	0	0	0
*12. Brucella canis* ATCC 23365	0	0	0
*13. Brucella melitensis* bv. 1 str. 16M	0	0	0
*14. Brucella ovis* ATCC 25840	0	0	0
*15. Brucella suis* 1330	0	0	0
*16. Chelativorans sp. BNC1*	1C	0	0
*17. Chelatococcus sp. CO‐6*	1C	0	1C
*18. Ensifer adhaerens OV14*	1C	1C	1C
*19. Hoeflea sp. IMCC20628*	1C	0	1C
*20. Martelella sp. AD‐3*	0	0	0
*21. Mesorhizobium ciceri* biovar biserrulae WSM1271	0	0	0
*22. Mesorhizobium loti* MAFF303099	0	1C	0
*23. Methylobacterium extorquens* AM1	5 C/3P	0	2P
*24. Methylobacterium extorquens* CM4	3C	0	2C
*25. Methylobacterium extorquens* DM4	1C	0	1C
*26. Methylobacterium nodulans* ORS 2060	1C/1P	0	1P
*27. Methylobacterium populi* BJ001	3C	0	2C
*28. Methylobacterium radiotolerans* JCM 2831	3C	0	0
*29. Methylobacterium* sp. 4‐46	1C	1C	0
*30. Neorhizobium galegae bv. officinalis bv. officinalis str. HAMBI 1141*	1C	0	C1
*31. Nitrobacter hamburgensis* X14	2P	1C	1C
*32. Ochrobactrum anthropi* DSM 6882	1C1/4C2	1C2	0
*33. Oligotropha carboxidovorans* OM4	1C/1P	0	0
*34. Oligotropha carboxidovorans* OM5	1C/2P	0	1P/3C
*35. Pelagibacterium halotolerans* B2	1C	0	0
*36. Rhizobium etli* bv. mimosae str. Mim1	0	0	0
*37. Rhizobium etli* CFN 42	0	0	1P
*38. Rhizobium etli* CIAT 652	0	0	1P
*39. Rhizobium leguminosarum* bv. trifolii WSM1325	0	0	0
*40. Rhizobium leguminosarum* bv. trifolii WSM2304	0	0	1P
*41. Rhizobium leguminosarum* bv. viciae 3841	0	0	1C
*42. Rhizobium tropici* CIAT 899	0	0	0
*43.Rhodopseudomonas palustris CGA009*	0	0	0
*44. Shinella sp. HZN7*	1	0	0
*45. Sinorhizobium fredii* NGR234	1P	0	0
*46. Sinorhizobium medicae* WSM419	1P	0	1C
*47. Sinorhizobium meliloti* 1021	2P	0	1C
*48. Sinorhizobium meliloti* 2011	1P	0	1C1
*49. Sinorhizobium meliloti* BL225C	2P	0	1C
*50. Sinorhizobium meliloti* GR4	3P	0	1C
*51. Sinorhizobium meliloti* Rm41	2P	0	1C
*52. Sinorhizobium meliloti* SM11	2P	0	1C
*53. Xanthobacter autotrophicus* Py2	2C	0	2P/1C
TOTAL	31C/24P	9C	24C/9P

a Replicons: C, Chromosome; C2, Chromosome number two: C3, Chromosome number three; CL, lineal chomosome; P, plasmid. Strains with CopZ, CopA and CusF homologs are yellow highlighted.

Since we could not retrieve CopZ homologs from *Rhizobium, Bradyrhizobium*, and *Mesorhizobium* genomes (Table S2 CopZ homologs), we searched for *S. pneumoniae* CupA homologs, which have been reported to functionally substitute CopZ in *Streptococcus* and *Lactobacillus*. CupA is a cell membrane‐anchored Cu^+^ chaperone that shares an isostructural cupredoxin‐like fold of 100 amino acids with the N‐terminus of *Streptococcus pneumoniae* CopA (Fu et al., 2013), which is absent in most CopA homologs. Our hmmsearch of Cupredoxin_1 family, PF13473, revealed that *S. pneumoniae* CupA homologs are mostly absent in the genomes of *Rhizobiales* (Table 5; Table S4 CupA homologs); however, we found eight homologous proteins encoded in the chromosome, five of them in strains without CopZ, including *Bradyrhizobium* and *Mesorhizobium,* and three in strains encoding CopZ. All putative CupA have a similar length (100–150 aa) and carry the cupredoxin_1 domain and the N‐terminal single transmembrane helix similar to CupA. The putative rhizobial CupA proteins and their respective CopA partners did not share the four amino acid, Cys….Cys‐X‐Met‐X‐Met motif, relevant for Cu(I) coordination and transfer, as observed in *S. pneumoniae* (Table S4 CupA homologs) (Fu et al., 2013).

The *E. coli* CusF is a small periplasmic copper chaperon protein predominantly found in gamma‐ and alpha‐proteobacteria with copper/silver resistance systems (Kim, Rensing, & McEnvoy, 2010). This protein is able to interact with CusB (part of the CusABC system) (Mealman et al., 2011, 2012) and CopA in *E. coli* (Padilla‐Benavides et al., 2014). We explored the presence of CusF in our data set (53 genomes), using the HMM‐profile available at the Pfam database for the CusF_Ec (PF11604) family. No CusF homologs were detected in 19 genomes of the *Rhizobiales* order; single copies were found in sixteen genomes, three *Methylobacterium* species contained two CusF copies. Three and four putative CusF were found in *X. autotrophycus* and *O. carboxydovorans*, respectively (Table 5; Table S3 CusF homologs). As members of the Ec_CusF family, most of the rhizobial homologs are small proteins ranging in size between 94 and 112 amino acids, which concurs with the 110 amino acids of Ec_CusF (Table S3 CusF homologs). Although all rhizobial CusF homologs conserve the invariable H36, M47 and M49 residues required for Cu^+^ binding (MBD), they present a significant polymorphism in the five electropositive residues (K23, K30, K31, H35 and R50) suggested as key elements in the interaction of Ec_CusF with Ec_CopA (Padilla‐Benavides et al., 2014) and Ec_CusF with CusB (Mealman et al., 2011, 2012) (Table S3 CusF homologs).

## 4 Discussion

A previous genome‐wide study on the trace element utilization of 540 bacterial genomes (Zhang & Gladyshev, 2010) revealed that two *Sinorhizobium* species, the only two rhizobia included, contained 22 copper‐dependent proteins, the largest bacterial cuproproteomes predicted to date. They also contained five Cu‐ATPases (CopA) with different functions and regulation (Patel et al., 2014). The high demand for copper suggested by these studies encouraged us to investigate the occurrence and diversity of three critical components of the Cu‐trafficking machinery: the P1B‐Cu ATPase CopA and the cytoplasmic and periplasmic chaperones, CopZ/CupA, and CusF, respectively, in other members of the *Rhizobiales* order.

### 4.1 P_1B_‐type ATPase divergence is higher than previously estimated

The use of profile HMM and maximum likelihood sequence analysis allowed us to refine the diversity of Cu‐ATPases beyond the established classification into P_1B‐1_ and P_1B‐3_‐ATPases. A similar approach has been proven to be effective for the discrimination of Cu‐specific protein subfamilies of sensors (Chang et al., 2014). Our results demonstrated that P_1B‐1_‐ATPase proteins have diverged into multiple subtypes. Even though our P_1B‐1_ ATPase dataset (410 sequences) was about half as large as that used in Smith's work employing Transitivity Clustering and Protein Similarity Network (672 sequences), our study displayed twice the number of inferred groups. We think that the higher diversity we revealed is due to the strategy employed: (1) Use of an unbiased selection of P_1B_‐ATPases from *Rhizobiales*, instead of limiting the search to characterized Cu‐ATPases; (2) The implementation of *ad hoc* profile HMM that includes the most divergent region of the P_1B_‐type ATPases located at the N‐terminal MBD; (3) The use of ML phylogenetic inference for sequence analysis instead of using BLAST‐ or ClustalW‐based methods; and (4) The analysis and allocation of 53 characterized P_1B_‐type ATPases in the phylogenetic tree, which allowed us to predict previously undefined groups of Cu‐ATPases with high certainty.

This scheme allowed us to discriminate different Cu‐ATPase subtypes and to uncover sequence‐specific motif changes, something difficult to achieve with previous data. One example is the subtype‐specific polymorphism in the N‐terminal MBD of subtypes P_1B‐1a_ and P_1B‐1b_, located in group VII, described in this work. Our results suggest that the N‐terminal domains of Cu‐ATPases behave as independent evolutionary units that have incorporated a number of new motifs, which potentially could modulate protein function. A similar evolutionary trend has been described in eukaryotic Cu‐ATPases (Gupta & Lutsenko, 2012).

### 4.2 Contribution of HGT to the diversity of CopA subfamilies found in the *Rhizobiales* order

The presence of *copA* genes on rhizobial plasmids proffers that these genes may be prone to spread through HGT. However, our analysis revealed that 25 of 30 genes predicted to be acquired through HGT were encoded in the chromosome and not in plasmids, as would be expected due to the large amount of extrachromosomal genetic information localized on plasmids (Table S7 G+G, CAI, GI). Interestingly, 12 of 22 chromosomally encoded putative *copA* genes belong to a gene cluster predicted to confer metal resistance; and three of 12 *copA* genes are part of predicted genomic islands. Fifty four percent of these foreign Cu‐ATPases belong to family VIII. *Methylobacterium* was the genus showing more foreign gene acquisition (14 putative Cu‐ATPases). All these data indicate that horizontal gene transfer has enriched the genomes of members of the *Rhizobiales* order with new Cu‐ATPase subtypes. The HGT of Cu‐ATPases seems to be independent of the replicon (plasmid or chromosome) where the genes are located. This is in agreement with previous studies that have reported HGT in rhizobia with mono‐replicon or multi‐replicon genome organization (Finan, 2002).

The HGT of P_1B_‐ATPases has also been detected in metal‐rich environments, supporting the contribution of HGT to the evolution of copper resistance (Bouzat & Hoostal, 2013; Martinez et al., 2006; Nongkhlaw, Kumar, Acharya, & Joshi, 2012).

Additionally, we found that 48% of the Cu‐ATPases surveyed in the *Rhizobiales* order are encoded in extrachromosomal replicons. This may partially explain the diversification of Cu‐ATPases observed in our phylogenetic analysis. According to the model of multipartite genomes, each replicon behaves as an independent evolutionary unit, encoding genes for specialized functions (diCenzo, MacLean, Milunovic, Golding, & Finan, 2014; Galardini, Pini, Bazzicalupo, Biondi, & Mengoni, 2013). Since plasmid copy number is usually higher than that of the chromosome, it may accelerate the evolution of plasmid‐borne genes (San Millan et al., 2016; Sano et al., 2014) In support of this hypothesis, in our ML‐phylogeny, we observed that chromosomally‐encoded FixI proteins, associated with cytochrome oxidases, form a small cluster in group XIV, whereas plasmid‐borne FixI1 and FixI2, with a different physiological role, were grouped in different clusters (Table S5 Cu‐ATPase subtypes).

### 4.3 What is the function of multiple and diverse Cu‐ATPases?

Our results show that the number and type of Cu‐ATPases is very variable among different members of the *Rhizobiales* order. Two experimentally testable hypotheses arise from these data. The first one proposes that the multiple Cu‐ATPases are functionally redundant and all of them act together to confer resistance when cells face a copper overload. If this hypothesis is true, the strains with high number of Cu‐ATPases should be adapted to live in a rich‐copper environment. The alternative hypothesis is that the presence of multiple Cu‐ATPases is the result of a genetic divergence process and each Cu‐ATPase confers copper resistance under different environmental conditions or in the different steps of interactions with their animal or plant host. In this regard, Patel et al. (2014) characterized mutants in each one of the five Cu‐ATPases of *S. meliloti* 2011. This phenotypic analysis revealed nonredundant copper resistance, *copA1* encodes the sole Cu‐ATPase required to detoxify the excess of copper in the cytoplasm. *Medicago sativa* plants inoculated with this mutant did not show nodule alterations. In contrast, plants inoculated with *S. meliloti* harboring *copA1b, fixI1, fixI2*, and *copA3* single mutations showed different abnormalities in nodule development. It is intriguing why other *Sinorhizobium* and *Rhizobium* species perform symbiotic nitrogen fixation with a minor number of Cu‐ATPases homologs. Are the differences in nodule organogenesis (determinate or indeterminate nodules) and nitrogen metabolism (the synthesis of amides or ureides) of host plants exerting selective pressure for the different types of ATPases? In the case of human pathogens, they have to deal with intracellular copper synthesized by macrophages to kill engulfed bacteria. The multiplicity of Cu‐ATPases may provide a resistance mechanism to escape from the human immune system? (Festa and Thiele, 2011). The answer to these questions will help us to understand the functional diversity of the Cu‐ATPases subtypes proposed in this study.

The presence of multiple Cu‐ATPases was first highlighted for pathogenic and symbiotic bacteria (Argüello, 2003; Argüello et al., 2011). In our study, we found multiple Cu‐ATPases in free‐living species of biotechnological interest due to their versatile metabolism as *Methylobacterium extorquens*,* Xanthobacter autotrophicus, Chelativorans sp*, and *Oligotropha carboxydovorans*, some of them were grouped together in subtype VIb. The role of multiple and diverse Cu‐ATPases in their metabolic properties remain to be elucidated.

### 4.4 Restricted occurrence of CopZ/CupA and CusF chaperones in the genomes of *Rhizobiales*

This study revealed the presence of multiple CopZ chaperones distributed in different replicons. In several species, the presence of numerous CopZ homologs correlates with the presence of multiple P1B‐Cu ATPases distributed in different replicons; for instance, eight CopZ proteins and five Cu‐ATPases are encoded in the genome of *M. extorquens* AM1 (Table S2 CopZ homologs), three *copZ* genes are located close to Cu‐ATPase encoding genes, other three are neighbors of mercuric, nucleotide, and metal transporters, and the last two are close to hypothetical proteins. These data suggest that putative CopZ metal binding proteins and CopA metal transporters are functionally linked and some of them may even have co‐evolved.

We found CopZ in four rhizobial genomes, previously surveyed in Ridge's work (Ridge, Zhang, & Gladyshev, 2008) but undetected by them; the same study was also unable to detect the presence of the CopZ homolog present in the *A. tumefaciens* plant pathogen which was characterized later on (Nawapan et al., 2009), indicating that Ridge′s method, based on BLAST searches, was not sensitive enough to detect distant CopZ homologs.The absence of CopZ homologs in fourteen rhizobial genomes from different species implies that an alternative protein should carry out its function. We found eight putative *S. pneumoniae* CupA homologs, five of them in strains without CopZ. However, putative CupA and their respective CopA partners did not share the Cys….Cys‐X‐Met‐X‐Met four amino acid motifs relevant for Cu^+^ coordination and transfer (Fu et al., 2013). These data suggest that different amino acid motifs are involved in coordination and transport, or a novel cytoplasmic chaperone may be present in those rhizobial species lacking CopZ or CupA homologs; both scenarios require to be tested These data are in agreement with a previous genome‐wide analysis based on BLAST searches that revealed that CopZ homologs predominate in the Firmicutes phylum, while being very scarce in all other bacterial phyla (Ridge et al., 2008). Ridge et al. (2008) also reported that CusF homologs were absent from the genomes of eighteen surveyed rhizobia. Searching 1,081 bacterial genomes for the presence of HWMM and HMMM metal‐binding motifs in a sequence alignment of the best BLASTP hits queried with *E. coli* K12 CusF (Kim et al. 2010), seven CusF‐like proteins were found in members of the *Rhizobiales* order. We reported twenty one putative rhizobial CusF proteins encoded in 12 rhizobial genomes, using the HMM‐profile of the CusF_Ec (PF11604) family; an unexpected diversity was observed in the protein length, which varies in a range from 94 to 236 amino acids, as well as in the polymorphism of the five electropositive residues (K2, K30, K31, H35 and R50) suggested as being critical for interaction with CopA and CusB. The location in plasmids of 40% of these CusF homologs may also be related to the protein evolution required to interact with the highly diversified group of P1B‐type ATPases. The absence of CusF in most rhizobial genomes leads us to hypothezise that alternative unknown chaperones distinct from CusF may be present in those rhizobia lacking CusF homologs.

Although our study is based on P_1B_‐type‐ATPases that confer resistance to metals, the analysis of multiplicity and diversity of transporters have general implications for the evolution of resistance, adaptation and innovation in bacteria. For instance, the co‐existence of antibiotics and metals in the environment has conducted to the co‐evolution of their mechanisms of resistance; the antibiotic resistance induced by exposure to toxic metals is well documented (Berg, Tom‐Petersen, & Nybroe, 2005; Chen et al., 2015; Stepanauskas et al., 2006). Furthermore, metals and antibiotic resistance genes tend to co‐occurr (Pal, Bengtsson‐Palme, Kristiansson, & Larsson, 2015; Su, Ye, & Zhu, 2012).

## 5 Conclusion

This study expands the occurrence of multiple and quite diverse Cu‐ATPases in the genomes of *Rhizobiales*. We propose a novel classification of Cu‐transporting ATPases in seven subfamilies, broadening the previous classifications of Cu^+^‐ and Cu^2+^‐ATPases in P_1B‐1_ and P_1B‐3_ subgroups.

The occurrence of copies of *copA* in plasmids or genomic islands as well as the exchange of Cu‐ATPases through lateral gene transfer depicts a genetic divergence process that bacteria have to follow to deal with the selective pressure exerted by copper in the environment.

The complete CopZ‐CopA‐CusF transport system found only in 37% of the analyzed rhizobial genomes, strongly suggests the existence of an alternative mechanism and/or unexplored proteins involved in copper transit.

This genome‐wide study of occurrence, diversity and evolution of Cu^+^‐ATPases in alpha‐proteobacteria belonging to the *Rhizobiales* order enriches our current knowledge, which has mainly been based on gamma‐proteobacteria analysis.

## Conflict of Interest

None declared.
