# Supplementary material for: A comprehensive phylogenetic analysis of copper transporting P1B ATPases from bacteria of the Rhizobiales order uncovers multiplicity, diversity and novel taxonomic subtypes
Source: Microbiologyopen. 2017 Feb 20;6(4):e00452. doi: 10.1002/mbo3.452 (PMC5552934; doi:10.1002/mbo3.452)
Supplement: Supplementary file 1 [file MBO3-6-na-s001.pdf]

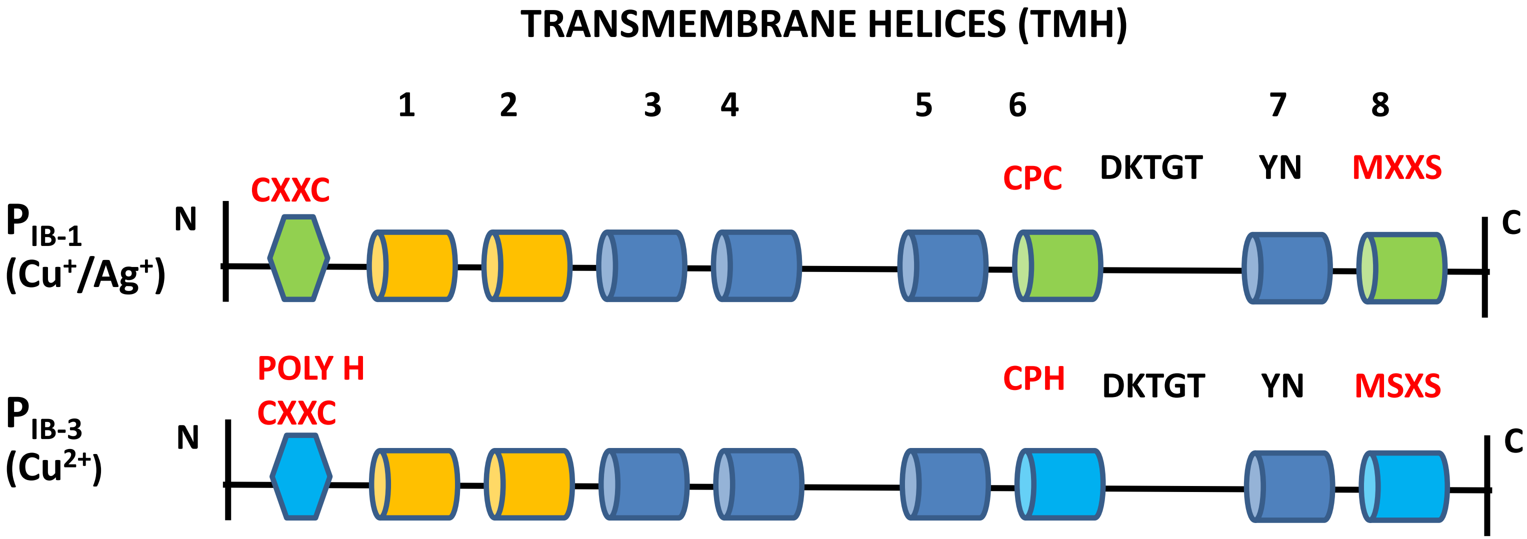

Fig. S1. Domain organization of P_{IB-1} and P_{IB-3} Cu-ATPases subtypes proposed by Argüello (2003, 2007). The N-terminal metal binding domain (MBD) and the metal-binding residues are indicated by a hexagon and amino acids in one letter code. The 8 transmembrane helices (TMH) and their strictly conserved residues are represented by cylinders and amino acids in one letter code. Residues in the TMH6 (CPC/CPH), TMH7 (YN) and TMH8 (MXXS/MSXS) are implicated in Cu^{+} or Cu^{2+} coordination. The aspartic residue (D) in the invariant DKTGT signature is the site of catalytic phosphorylation. Residues in red are conserved signature motifs conserved in most of P_{IB-1} and P_{IB-3} Cu-ATPases.
