# Supplementary material for: A comprehensive phylogenetic analysis of copper transporting P1B ATPases from bacteria of the Rhizobiales order uncovers multiplicity, diversity and novel taxonomic subtypes
Source: Microbiologyopen. 2017 Feb 20;6(4):e00452. doi: 10.1002/mbo3.452 (PMC5552934; doi:10.1002/mbo3.452)
Supplement: Supplementary file 2 [file MBO3-6-na-s002.pdf]

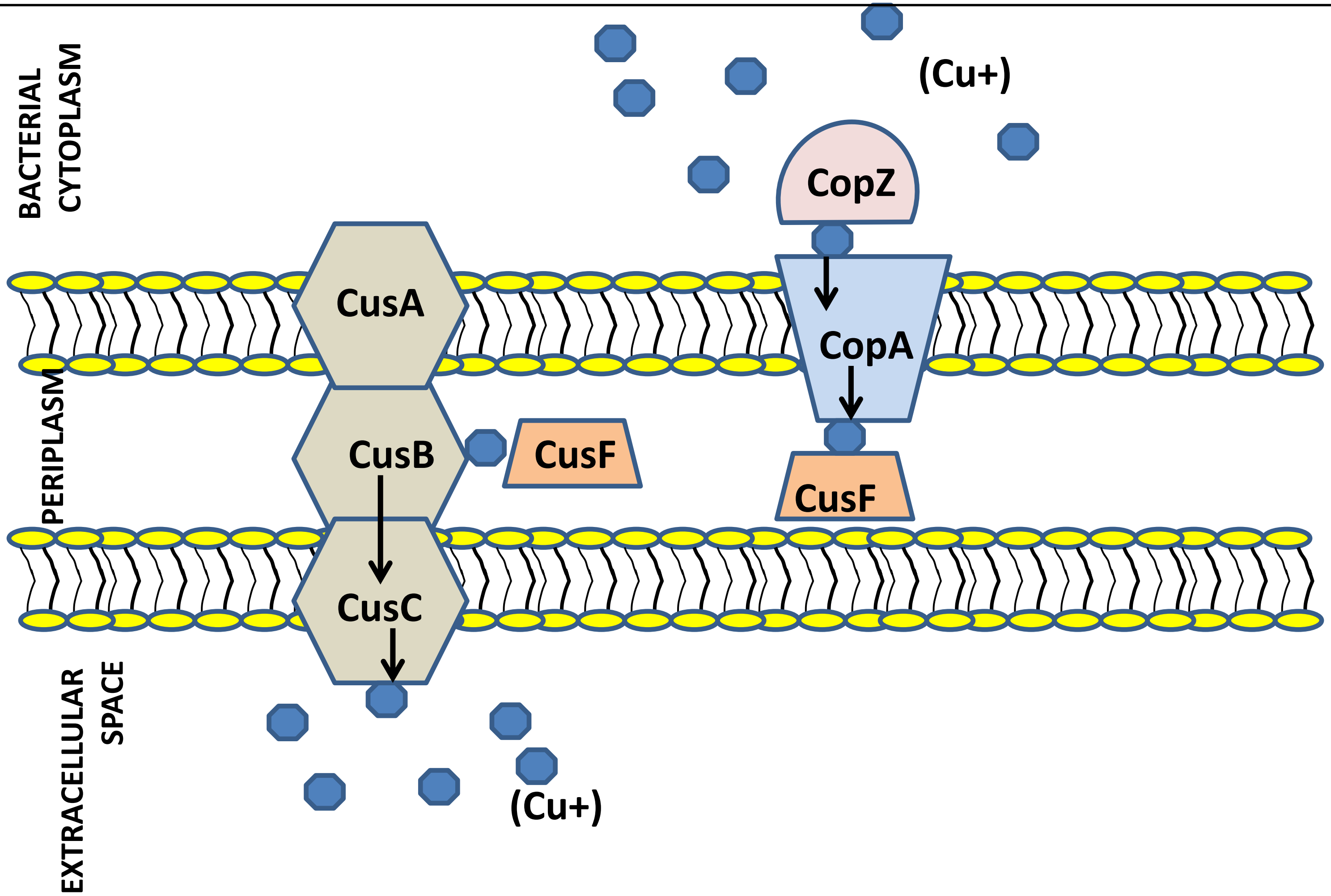

Fig.. S2. Model of cytoplasmic copper efflux through the CopZ-CopA-CusF transport system. CopZ is a soluble cytoplasmic chaperone protein that delivers Cu^+ to the trans-membrane Cu^+-efflux ATPase CopA. Subsequently Cu^+ is transferred from CopA to CusF, a soluble periplasmic protein that delivers Cu^+ to the CusABC export system, which exports Cu^+ to the extracellular space.
