# Supplementary figures and images for: A comprehensive phylogenetic analysis of copper transporting P1B ATPases from bacteria of the Rhizobiales order uncovers multiplicity, diversity and novel taxonomic subtypes
Source: Microbiologyopen. 2017 Feb 20;6(4):e00452. doi: 10.1002/mbo3.452 (PMC5552934; doi:10.1002/mbo3.452)

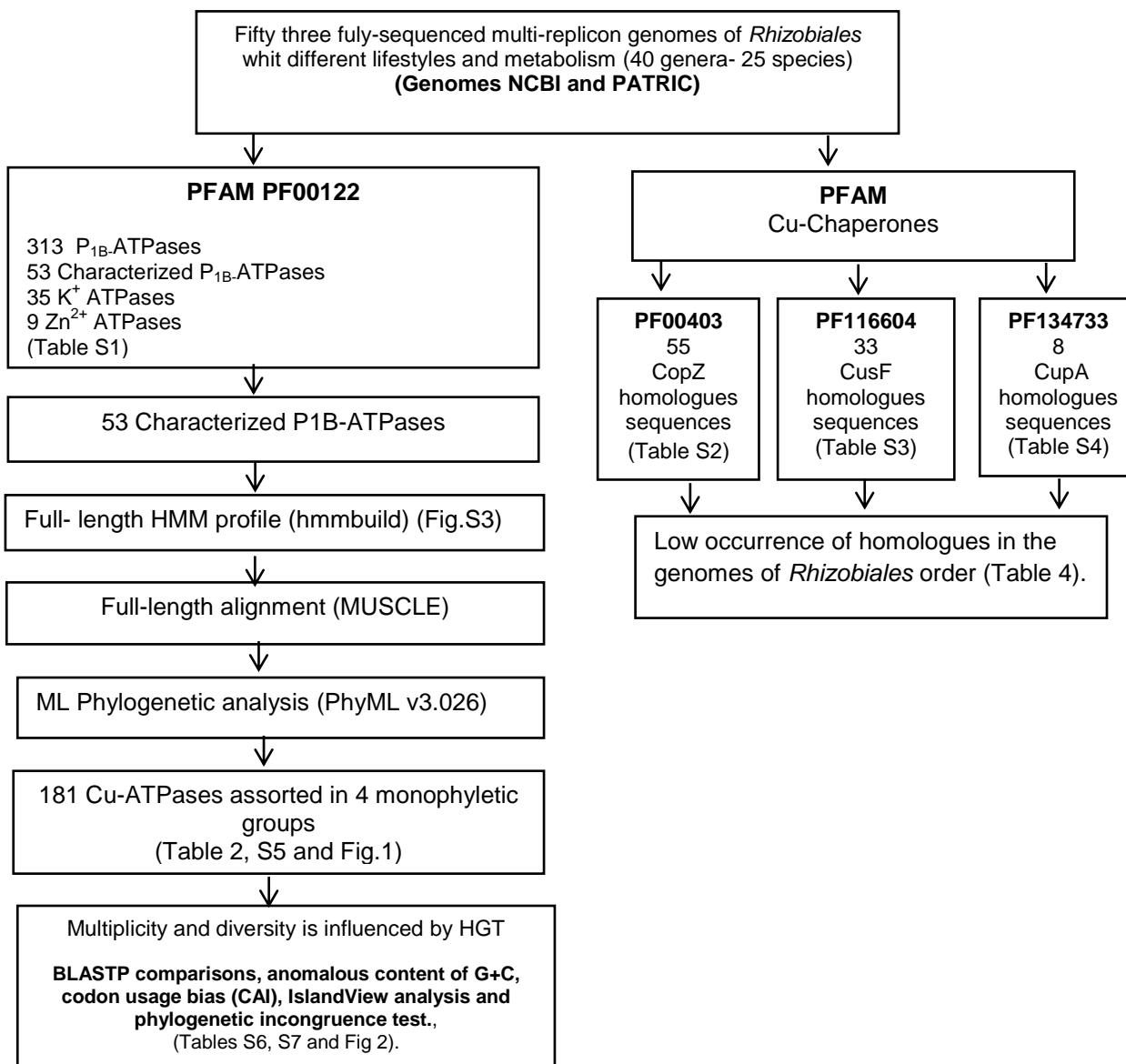

Figure S3. Diagram of data sources (in bold) and phylogenetic analysis.

Supplement: Supplementary file 3 [file MBO3-6-na-s003.pdf]

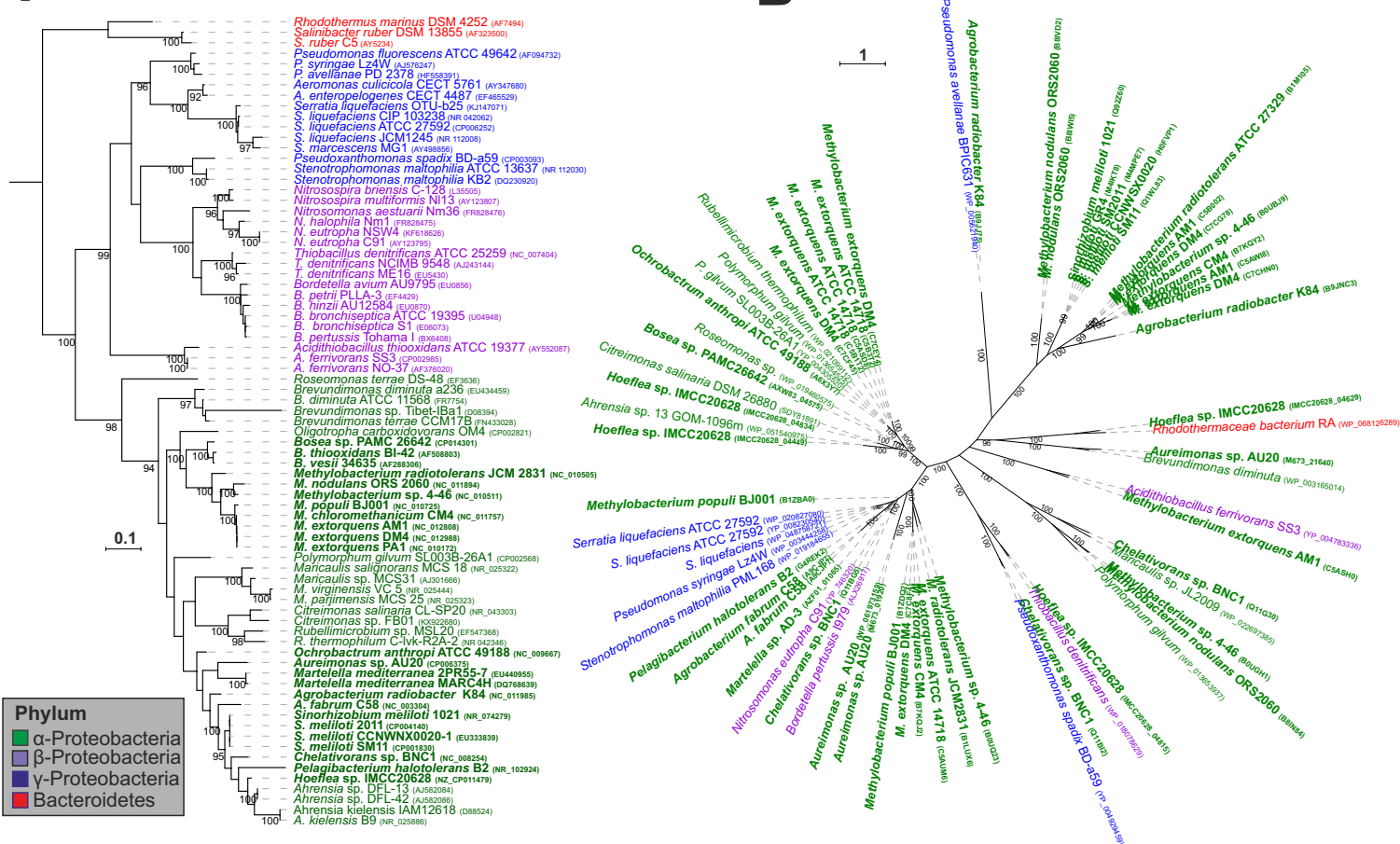

Supplement: Supplementary file 6 [file MBO3-6-na-s006.pdf]
