# Supplementary material for: A comprehensive phylogenetic analysis of copper transporting P1B ATPases from bacteria of the Rhizobiales order uncovers multiplicity, diversity and novel taxonomic subtypes
Source: Microbiologyopen. 2017 Feb 20;6(4):e00452. doi: 10.1002/mbo3.452 (PMC5552934; doi:10.1002/mbo3.452)
Supplement: Supplementary file 4 [file MBO3-6-na-s004.pdf]

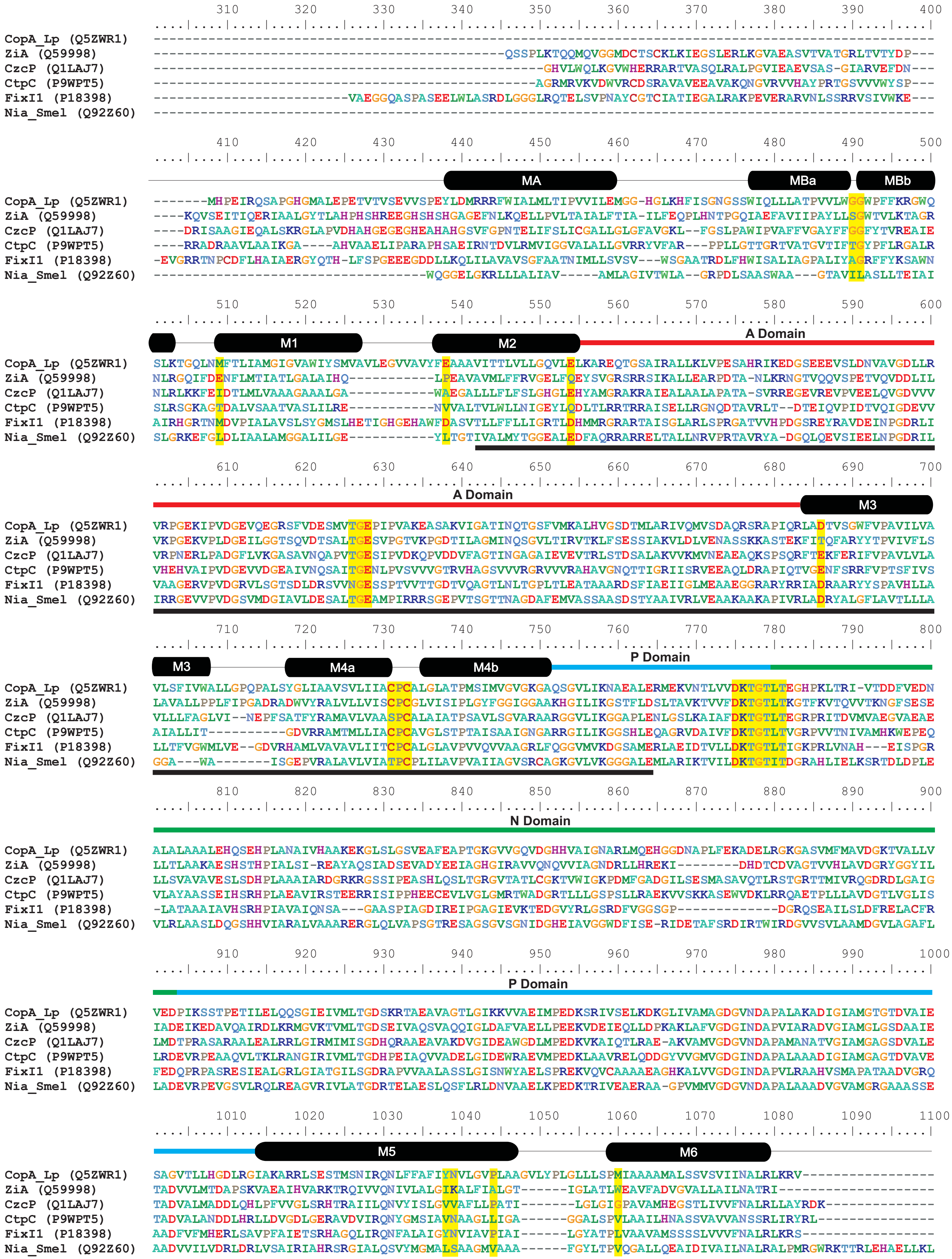

Fig. S4. Alignment of PIB-ATPases used to infer a new HMM profile from 53 characterized PIB-type ATPases. Six of them having Cu (CopA, FixI1), Zn (ZiaA), Co (CzcP),Fe, Ni (Nia) and Mn (CtpC) as substrates are shown.The A- (actuator), P- (phosphorylation), N- (nucleotide binding) and the transmembrane helices (black cylinders) are also indicated based on the crystal CopA structure (Gourdon et al 2011).The black underlined residues represent the profile HMM available at Pfam.
