# Supplementary material for: A comprehensive phylogenetic analysis of copper transporting P1B ATPases from bacteria of the Rhizobiales order uncovers multiplicity, diversity and novel taxonomic subtypes
Source: Microbiologyopen. 2017 Feb 20;6(4):e00452. doi: 10.1002/mbo3.452 (PMC5552934; doi:10.1002/mbo3.452)
Supplement: Supplementary file 5 [file MBO3-6-na-s005.pdf]

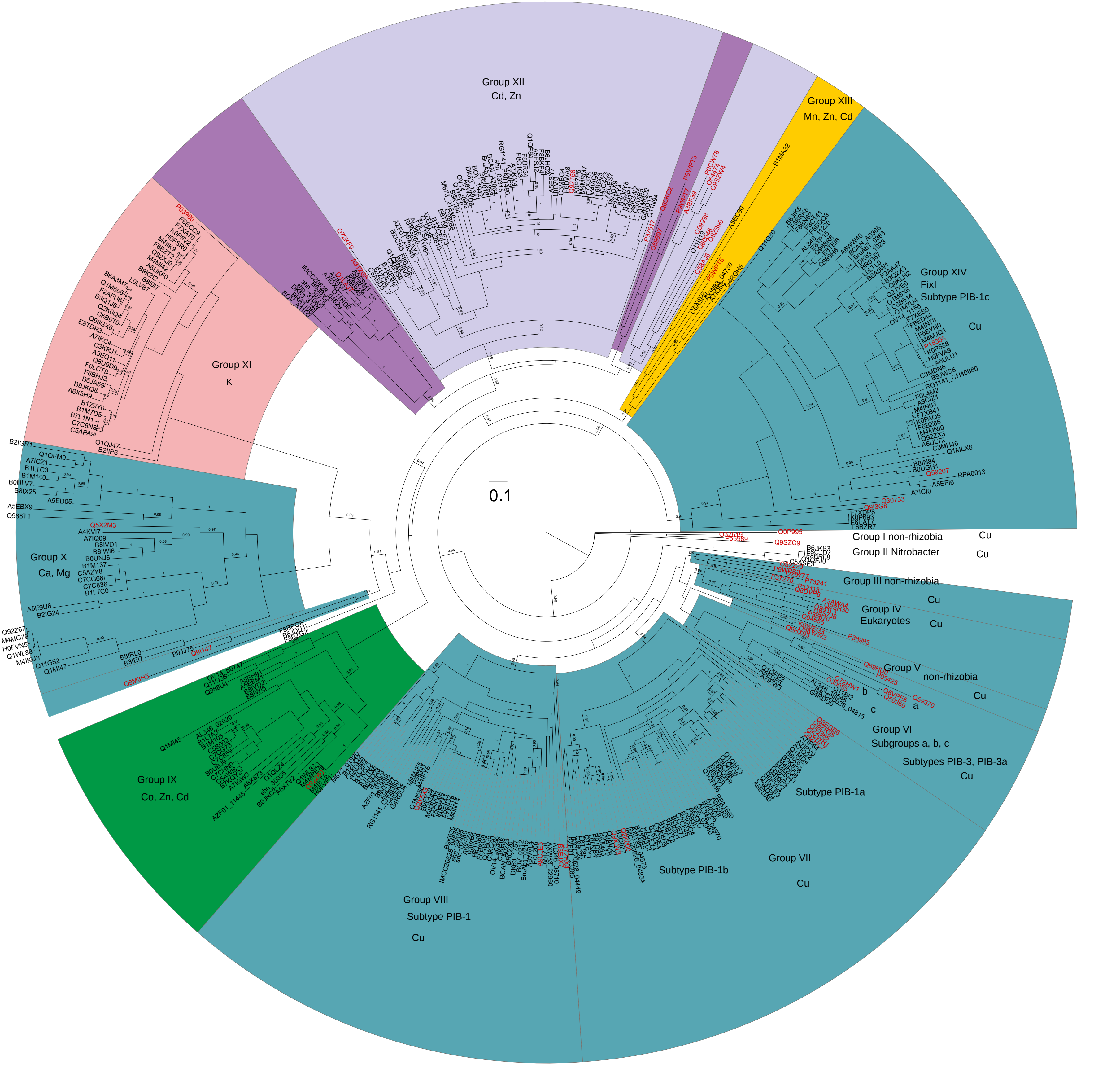

Fig S5. Diversity of P_{IB}-type ATPases revealed by ML-Phylogeny. The ML phylogeny was built by using a 410 P_{IB}-ATPase protein alignment. The fourteen monophyletic groups and their subtypes with a branch support (SH-like) p value ≥ 0.9 are indicated by Roman numbers (I to XIV). Rhizobial Cu-ATPases and their subtypes were distributed in four subfamilies (VI, VII, VIII, XIV). Argüello´s subtypes PIB-1 and PIB-3 are grouped in monophyletic groups VI, VII and VIII. Novel subtypes were distributed in three monophyletic groups (VII, VIII and XIV). The evolutionary relationships of Cu-ATPases with PIB-ATPases for Zn, Cd, Co, Mn and Mg are also shown. The P-ATPases for potassium (K) was used as outgroup.
