# Supplementary material for: A comprehensive phylogenetic analysis of copper transporting P1B ATPases from bacteria of the Rhizobiales order uncovers multiplicity, diversity and novel taxonomic subtypes
Source: Microbiologyopen. 2017 Feb 20;6(4):e00452. doi: 10.1002/mbo3.452 (PMC5552934; doi:10.1002/mbo3.452)
Supplement: Supplementary file 14 [file MBO3-6-na-s014.docx]

**Table S8. Occurrence of putative CopA in 53 Rhizobial multireplicon genomes.***

|  | **Established**  **CopA Subtypes** | | **Novel CopA Subtypes** | | | |  |
| --- | --- | --- | --- | --- | --- | --- | --- |
| **STRAINS** | **P_1B-1_** | **P_1B-3_** | **P_1B-1a_** | **P_1B-1b_** | **P_1B-1c_** | **P_1B-3a_** | **Total** |
| *1. Agrobacterium fabrum* str. C58 | 1c | - | - | - | - | - | **1c** |
| *2. Agrobacterium radiobacter* K84 | - | - | - | 1c | - | - | **1c** |
| *3. Agrobacterium* sp. H13-3 | 1c | - | - | - | - | - | **1c** |
| *4. Agrobacterium vitis* S4 | 1p 1c | - | 1p | - | 1c | - | **2p 2c** |
| *5.Aureimonas sp. AU20* | 1c | - | - | - | - |  | **1c** |
| *6. Bartonella grahamii* as4aup | - | - | - | - | - | - | **0** |
| *7. Bartonella tribocorum* CIP 105476 | - | - | - | - | - | - | **0** |
| *8. Beijerinckia indica* subsp. indica ATCC 9039 | 1c | - | 1c | 1c | - | - | **3c** |
| *9. Bosea sp. PAMC 26642* | 1c | - | - | 1c | - | - | **2c** |
| *10. Bradyrhizobium* sp. BTAi1 | - | 1p | 1p 2c | - | 1c | 1p | **3p 3c** |
| *11. Brucella abortus bv. 1 str. 9-941* | 1c | - | - | - | 2c | - | **1p 2c** |
| *12. Brucella canis ATCC 23365* | 2c | - | - | - | - | - | **2c** |
| *13.* Brucella melitensis bv. 1 str. 16M | 1c | - | - | - | - | - | **1c** |
| *14.* Brucella ovis ATCC 25840 | 1c | - | - | - | - | - | **1c** |
| *15. Brucella suis 1330* | 1c | - | - | - | - | - | **1c** |
| *16. Chelativorans* sp. BNC1 | 1c | 1c | 1c | 1c | - | - | **4c** |
| *17. Chelatococcus sp. CO-6* | 1c | 1c | 1c | - | 2c | - | **5c** |
| *18. Ensifer adhaerens OV14* | 1c | - | - | - | - | - | **1c** |
| *19. Hoeflea sp. IMCC20628* | 1p | 1p | - | 1c 1p | - | - | **3p 1c** |
| *20. Martelella sp. AD-3* | 1c | - | - | 1p | - | - | **1c 1p** |
| *21. Mesorhizobium ciceri* biovar biserrulae WSM1271- | - | - | - | 1c | 1p 1c | - | **1p 2c** |
| *22. Mesorhizobium loti* MAFF303099 | - | - | - | 1p | 2c | - | **1p 2c** |
| *23. Methylobacterium extorquens* AM1 | 1c | - | 2p 1c | - | - | - | **2c 2p** |
| *24. Methylobacterium extorquens* CM4 | 1c |  | 2c | - | - | - | **3c** |
| *25. Methylobacterium extorquens* DM4 | 1c | - | 2c | - | - | - | **3c** |
| *26. Methylobacterium nodulans* ORS 2060 | 1p | - | 1c 1p | - | 1c | - | **2c 2p** |
| *27. Methylobacterium populi* BJ001 | 2c | - | 2c | - | - | - | **4c** |
| *-28. Methylobacterium radiotolerans* JCM 2831 | 1c | - | - | - | - | - | **1c** |
| *29. Methylobacterium* sp. 4-46 | 1c | - | 1c | - | 1c | - | **3c** |
| *30. Neorhizobium galegae* HAMBI1141 | 1c | - | - | - | - | - | **1c** |
| *31. Nitrobacter hamburgensis* X14 | - | 1p | 2p 1c | - | - | 1p | **4p 1c** |
| *32. Ochrobactrum anthropi* DSM 6882 | 1c | - | 1c | 2c | 2c | - | **6c** |
| *33.Oligotropha carboxidovorans* OM4 | 1c | - | 1c 1p | - | - | - | **2c 1p** |
| *34. Oligotropha carboxidovorans* OM5 | 1c | - | 1c 1p | 2p | 2c 2p | - | **4c 5p** |
| *35. Pelagibacterium halotolerans* B2 | 2c | 1c | - | - | - | - | **3c** |
| *36. . Rhizobium etli* CFN 42 | - | - | - | 1p | 3p | - | **4p** |
| *37 Rhizobium etli* CIAT 652 | - | - | - | 1p | 1p | - | **2p** |
|  |  |  |  |  |  |  |  |
|  | **Established**  **CopA Subtypes** | | **Novel CopA Subtypes** | | | |  |
| **STRAINS** | **P_1B-1_** | **P_1B-3_** | **P_1B-1a_** | **P_1B-1b_** | **P_1B-1c_** | **P_1B-1d_** | **Total** |
| *38. Rhizobium leguminosarum* bv. trifolii WSM1325 | - | - | - | 1p | - | - | **1p** |
| *39. Rhizobium leguminosarum* bv. trifolii WSM2304 | - | - | - | 1p | 1p | - | **2p** |
| *40. Rhizobium leguminosarum* bv. viciae 3841 | 1p | - | 1c | 1p | 3c 2p | - | **4p 4c** |
| *41. Rhizobium tropici* CIAT 899 | - | - | - | 1c | 1p | - | **1c 1p** |
| *42. Rhodopseudomonas palustris* CGA009 | - | - | 1c | - | 1c | - | **2c** |
| *43. Shinella sp*. HZN7 | 1p 1c | - | 1c | - | - | - | **1p 1c** |
| *44. Sinorhizobium fredii* NGR234 | 2p | - | 1p | - | 3c | - | **3p 3c** |
| *45. Sinorhizobium medicae* WSM419 | 1p | - | - | - | 2p | - | **3p** |
| *46.Sinorhizobium meliloti* 1021 | 2p | - | 1p | - | 2p | - | **5p** |
| *47. Sinorhizobium meliloti* 2011 | 2p | - | 1p | - | 2p | - | **5p** |
| *48.Sinorhizobium meliloti* AK83 | - | - | - | - | - | - | **Draft** |
| *49.Sinorhizobium meliloti* BL225C | 1p | - | - | - | 3p | - | **4p** |
| *50. Sinorhizobium meliloti* GR4 | 1p | - | 3p | - | 2p | - | **6p** |
| *51. Sinorhizobium meliloti* Rm41 | 1p | - | - | - | 2p | - | **3p** |
| *52. Sinorhizobium meliloti* SM11 | 1p | - | 1p | - | 3p | - | **5p** |
| *53. Xanthobacter autotrophicus* Py2 | - | 1p | 2p 2c | - | 1c | 1p | **4p 4c** |
| TOTAL | C31 P16 | C3 4P | C23 P18 | C9 P10 | C23 P27 | C0 P3 |  |
|  | 47 | 7 | 41 | 19 | 50 | 3 |  |

*Replicons: C, Chromosome; C2, Chromosome number two: C3, Chromosome number three; CL, lineal chomosome; P, plasmid.
